# Shear and Interface Properties for Unidirectional, Woven, and Hybrid M21 Particle-Toughened Composites

**DOI:** 10.3390/ma19030540

**Published:** 2026-01-29

**Authors:** Andrew Seamone, Anthony Waas, Vipul Ranatunga

**Affiliations:** 1 Department of Aerospace Engineering, University of Michigan, Ann Arbor, MI 48109, USA; 2 School for Engineering of Matter, Transport and Energy, Arizona State University, Tempe, AZ 85287, USA; 3 AFRL, Wright-Patterson AFB, Dayton, OH 45433, USA

**Keywords:** carbon fiber reinforced polymer (CFRP), composite shear behavior, double cantilever beam (DCB), end-notch flexure (ENF), finite element analysis (FEA), design of experiments (DOE), failure analysis, damage mechanics

## Abstract

The M21 epoxy matrix is a toughened material designed to enhance the fracture resistance of carbon fiber-reinforced polymers (CFRPs). This study presents an experimental characterization of the shear and interlaminar properties required for validating computational damage models of hybrid laminated composite panels manufactured with the M21 material system. In-plane shear behavior was evaluated using ±45 (PM45) tests, while interlaminar fracture properties were characterized through double cantilever beam (DCB) and end-notched flexure (ENF) tests. The results demonstrate that hybrid laminates exhibit high interfacial fracture toughness, with notably increased resistance observed in woven–woven and unidirectional–woven interface pairs. Parametric studies identified cohesive strength and fracture energy as the dominant parameters governing delamination behavior in numerical simulations. Corresponding values were extracted for each interface type, enabling accurate representation of damage initiation and propagation in finite element models. To the authors’ knowledge, this work provides the first experimental dataset for the listed M21-based hybrid unidirectional–woven and woven–woven interfaces, establishing a benchmark for future modeling and simulation of toughened composite structures.

## 1. Introduction

Composite materials, particularly carbon fiber-reinforced polymers (CFRPs), are increasingly used in modern aerospace applications due to their superior strength-to-weight ratio, physical property tailorability, and fatigue resistance. Among these, particle-toughened epoxy systems such as the M21 matrix have emerged as promising candidates for structural components where both strength and durability are critical. The introduction of toughened interlayers improves crack resistance and energy absorption, yet it also introduces complexities in understanding and predicting failure mechanisms across different composite architectures.

Accurate modeling of composite behavior requires detailed knowledge of both shear nonlinearity and interfacial fracture toughness, as these phenomena govern the initiation and growth of damage under operational loading [1,2,3,4,5]. Micro-scale processes such as hackling, shear banding, and fissure formation reduce shear stiffness and alter laminate response, while interface properties dictate whether delamination progresses in a stable or unstable manner [6,7,8]. As aerospace and defense applications look to innovate with hybrid composite layups (where unidirectional tape is combined with woven architectures), it becomes essential to characterize how these microstructural features influence shear response and interfacial toughness.

This study addresses these challenges by systematically investigating the in-plane shear and interface (between plies) properties of unidirectional, woven, and hybrid M21 particle-toughened composites. A series of plus–minus 45° (PM45) shear tests, double cantilever beam (DCB) experiments, and end-notch flexure (ENF) tests were conducted to quantify nonlinear in-plane shear behavior and to establish mode I and mode II interlaminar fracture toughness values across various layup configurations. Previous studies have facilitated the analysis of material and system properties from these experiments, and this work builds on these strategies for the newest composite materials [9,10,11,12,13,14]. The experimental findings are complemented by finite element models using enhanced Schapery theory and cohesive zone modeling to identify key parameters governing crack propagation. Together, these efforts aim to provide a validated foundation for predictive simulation of hybrid composite structures, enabling a more reliable design of next-generation aerospace components.

One of the most critical aspects of composite materials for successful modeling is matrix shear nonlinearity, which arises from micro-scale damages such as hackling, shear banding, and micro-fissures [15,16]. Microdamages occurring in the matrix of composite structures drive the degradation of matrix-related properties of laminates which lowers the transverse and shear moduli of the material. In order to understand these damage mechanisms, plus–minus 45 (PM45) experiments are presented in this section along with the process of identifying the appropriate parameters to incorporate this into numerical predictive models.

Enhanced Schapery theory (EST) is a thermodynamically based work potential that combines Schapery theory (which captures the nonlinearity of the pre-peak region in the stress–strain curve) with Bazant’s crack band (CB) approach (which captures post-peak degradation of fiber and matrix damage). EST is implemented through the use of a VUMAT in Abaqus which allows for a custom definition of the mechanical constitutive behavior of a material.

**Figure 1:**
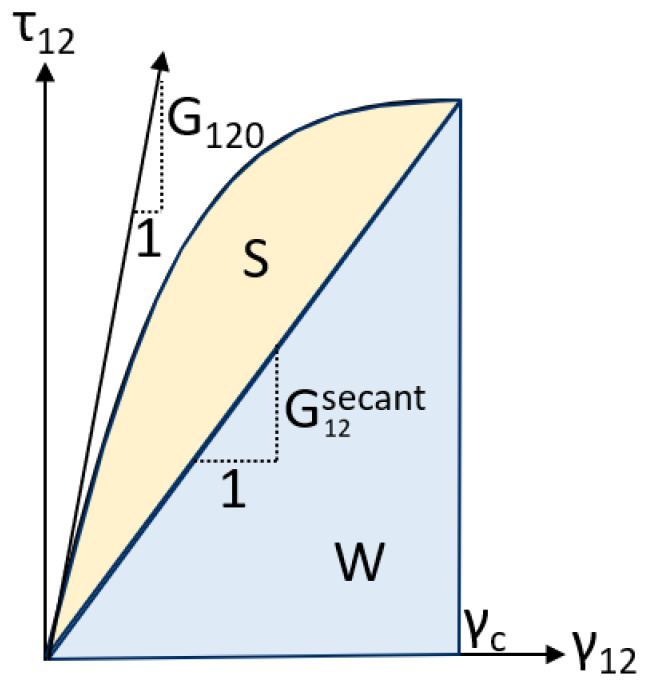
Pre-peak nonlinearity in EST.

Figure 1 presents the energy dissipation in a simple manner, in which energy dissipation due to micro-scale cracking is represented by area *S* and the strain energy density is represented by *W*. The material has an initial shear modulus of $G_{120}$ and a secant shear modulus $G_{12}^{sec}$ which decays until the material reaches a failure criteria strain $\gamma_{12c}$ to enter the crack band. The process of calculating the EST behavior is well documented in several prior studies, and the equations can be seen in these studies [5,15,17,18,19,20,21,22].

The DCB and ENF experiments are well-studied methods to characterize fracture toughness in composite materials. These tests can be used to determine the amount of energy required for a crack to propagate on a specified interface of a composite material. The M21 composite material is an interface-toughened system investigated in this study. Prior studies have investigated toughening strategies of similar systems [23,24]. The growth of the cracks along the interfaces of these types of composites tends to be unstable, indicated by non-smooth load versus displacement responses. It is important to understand the mechanisms in which cracks grow in a stable or unstable manner [25]. Previous studies have investigated the fracture toughnesses for traditional interfaces where both lamina surrounding the crack are unidirectional tape [26,27]. Additional studies have also quantified woven and hybrid interfaces, in which one or both interfaces adjacent to the artificial crack consist of a woven twill lamina [28,29,30]. The fracture toughnesses in these cases are much higher, resulting from the microstructure of the interface. Traditional tape laminae allow avenues for the crack to propagate between fibers, while the local changes to the microstructure of woven lamina require higher amounts of energy to propagate a crack.

This study evaluates the results of the DCB and ENF experiments and includes experiments from two previous studies [28,31]. In addition to these, additional experiments were performed to characterize new woven and hybrid interfaces that are of interest for future applications. Finite element models were developed to validate the fracture toughnesses in a numerical setting and to identify the required cohesive strengths to accurately model failure. The data presented in this report provide a foundation for the characterization of the M21 material system’s important and complex parameters. This study aims to aid future modeling efforts that utilize woven and hybrid interfaces by identifying the cohesive strengths and fracture toughnesses needed for cohesive modeling.

The rationale for studying these properties for the M21 resin system was to deepen our understanding of shear and interfacial behavior of particle-toughened systems. The M21 system was selected as a candidate material for future airframe applications as part of an AFRL research project, and this work assists in the development of numerical tools that better inform the design process.

## 2. Shear Characterization Study

### 2.1. Specimen Description

**Figure 2:**
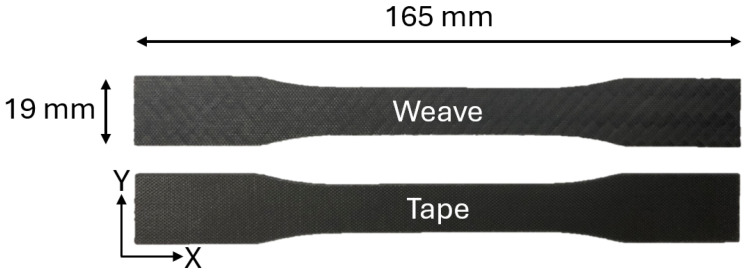
A woven and tape plus–minus 45 specimen used for characterization of the nonlinearity in the shear modulus of the matrix.

Eight tape specimens and eight woven specimens were tested in the shear experiment. Each dog-bone specimen was 165 mm in length and 19 mm in width and is depicted in Figure 2. The cross section tapers down to a width of 13 mm in the center of the specimen. The geometry of the specimen was decided based on standard ASTM D638 [32].

The tape specimens were made up of eight unidirectional plies and the woven specimens contained four woven plies. The layups and sample thicknesses are shown in Table 1. The *W* in the layup column represents a woven lamina.

### 2.2. PM45 Experiment

The plus–minus 45 experiment allows for the shear response of the tape and woven laminates to be characterized.

#### 2.2.1. Experimental Method

**Figure 3:**
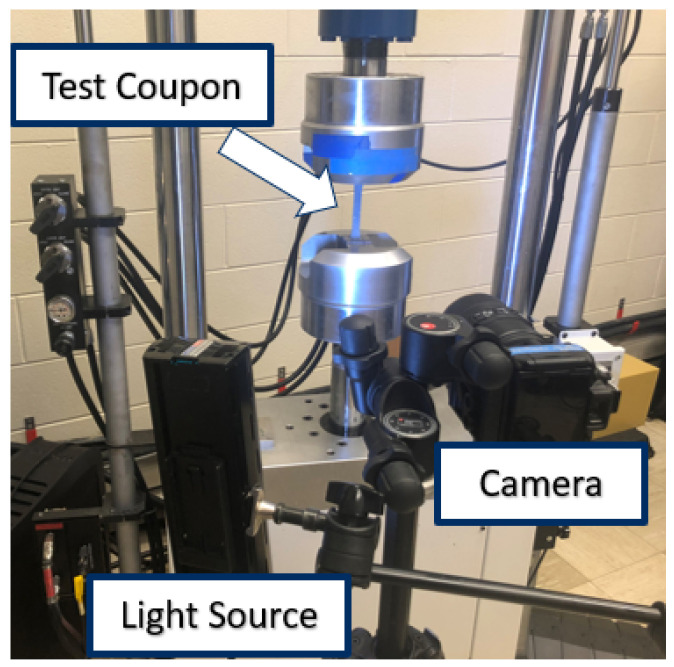
Test setup for the plus–minus 45 experiment.

The experiment was performed by loading the test specimen in tension using a Shorewestern load frame. The experiment was displacement-controlled with loading rates of 1.0 mm/min, 5.0 mm/min, and 10.0 mm/min used for different specimens. These three rates were selected to investigate if there was a noticeable difference in the shear stress–strain relationship in the quasi-static loading rate realm. Load data was recorded from the test frame. Displacement and strain data were gathered with the use of 2D Digital Image Correlation (DIC) using the GOM Correlate software. The test specimens were spray-painted matte white and speckled with matte black paint to create a pattern suitable for 2D DIC analysis. Images of the specimen were taken using a Nikon camera at one-second intervals, and an LED provided sufficient illumination to the sample. A depiction of the test setup is shown in Figure 3.

#### 2.2.2. Experimental Results

**Figure 4:**
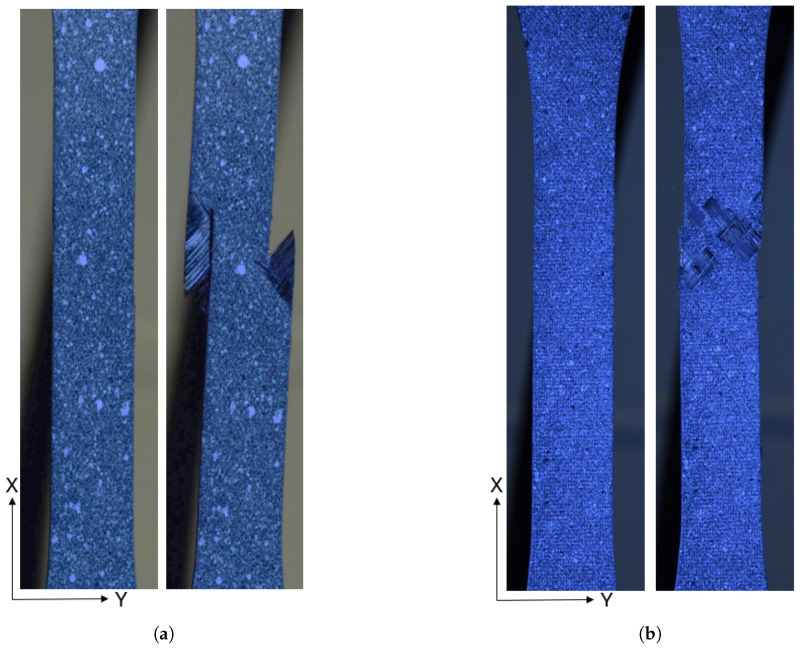
PM45 shear stress versus shear strain relationship. (**a**) A tape PM45 specimen before and after failure. (**b**) A woven PM45 specimen before and after failure.

The tape specimen in Figure 4a shows the speckle pattern used to acquire the displacement and strain information. A failure feature occurs parallel to the fiber direction. The woven specimen is shown in Figure 4b. The failure does not occur along the fiber direction, rather around the tow boundaries and perpendicular to the loading direction. The break is not clear as in the case of the tape failure.

**Figure 5:**
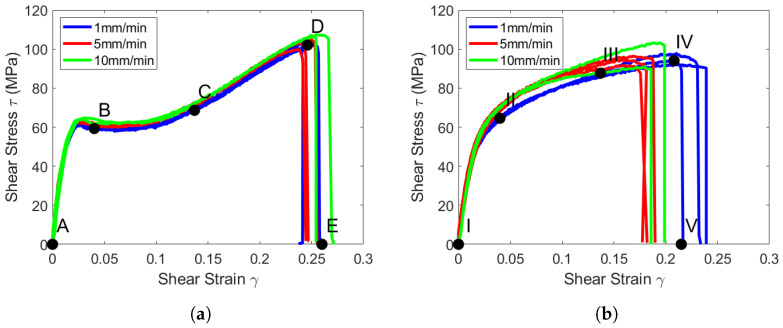
PM45 shear stress vs. shear strain relationship. (**a**) Shear stress vs. shear strain response for the tape samples at quasi-static loading rates. (**b**) Shear stress vs. shear strain response for the woven samples at quasi-static loading rates.

The experimental data for the plus–minus 45 tests are shown in Figure 5.

The tape specimens in Figure 5a shows a typical response for a toughened interface composite [33,34]. During the initial loading portion, the stiffness of the composite is high, and damage begins to accumulate in the form of micro-cracking within the material. The plateau region represents the loading stage where macro-cracks begin to form. The final increase in load represents a progressive delamination progress between laminae until a failure load is reached. These results apply only to the quasi-static range of 1.0–10.0 mm/min.

**Figure 6:**
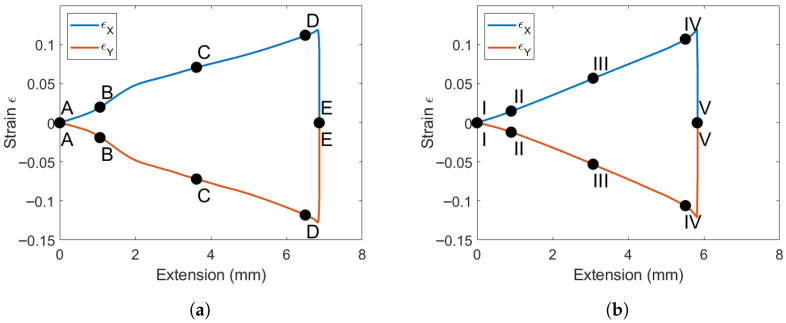
Strain vs. extension relationship for the matrix-toughened M21 PM45 specimens. (**a**) Tape shear response. (**b**) Woven shear response.

The loading direction $\epsilon_{x}$ and transverse $\epsilon_{y}$ strains are shown for the tape and woven laminates in Figure 6. The tape response in Figure 6a shows a kink in the strain measurement. This was consistent for all tape samples and occurs in matrix-toughened layups. This kink is not observed in the woven laminate. A possible explanation for the difference in these responses is the saturation of micro-cracks within the structure. The load plateaus in the tape specimens correspond to the micro-cracks saturating and allow the structure as a whole to relax slightly, which creates a temporary increase in $\epsilon_{x}$. The same change is seen in $\epsilon_{y}$, as these strains are proportional.

**Figure 7:**
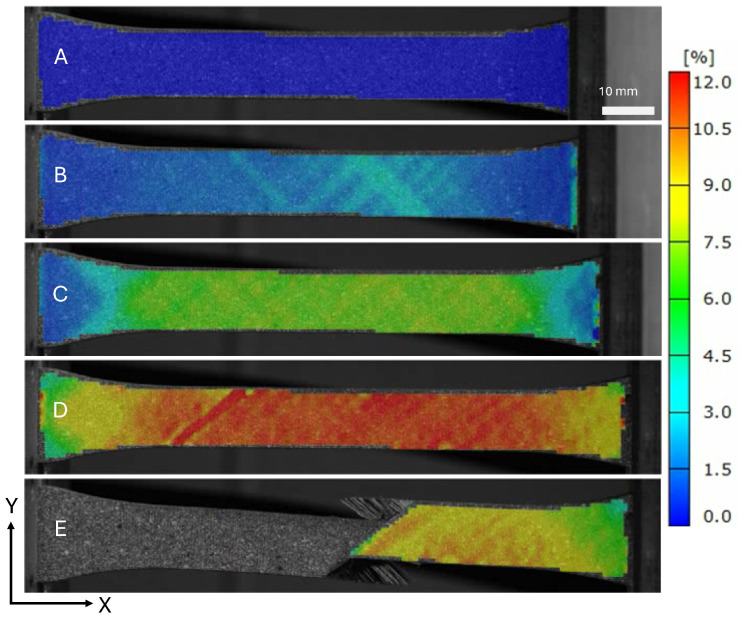
Surface loading strains in the loading direction $\epsilon_{x}$ of a tape PM45 specimen.

**Figure 8:**
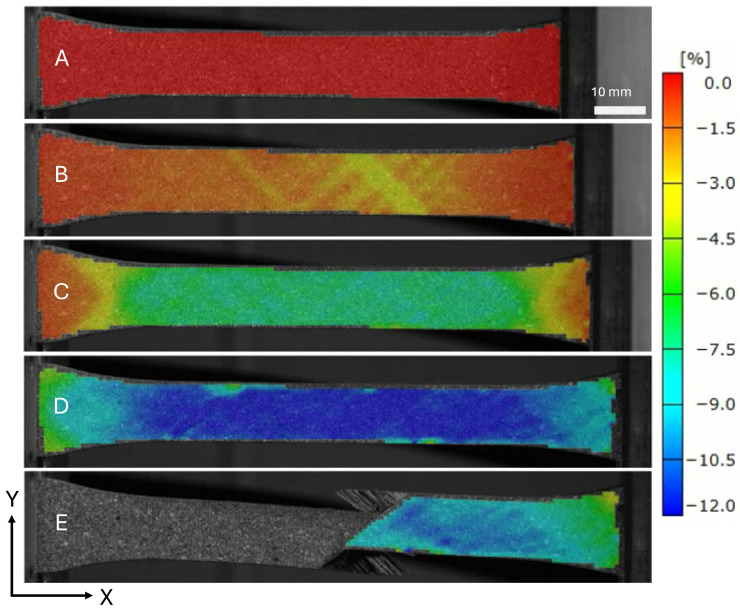
Surface loading strains in the loading direction $\epsilon_{y}$ of a tape PM45 specimen.

The strain field at the indicated locations from Figure 5 are shown in Figure 7 and Figure 8. The saturation of micro-cracks in the structure can be observed between points B and C. Point D shows the specimen prior to ultimate failure, and Point E shows when the structure has lost load-carrying capability.

**Figure 9:**
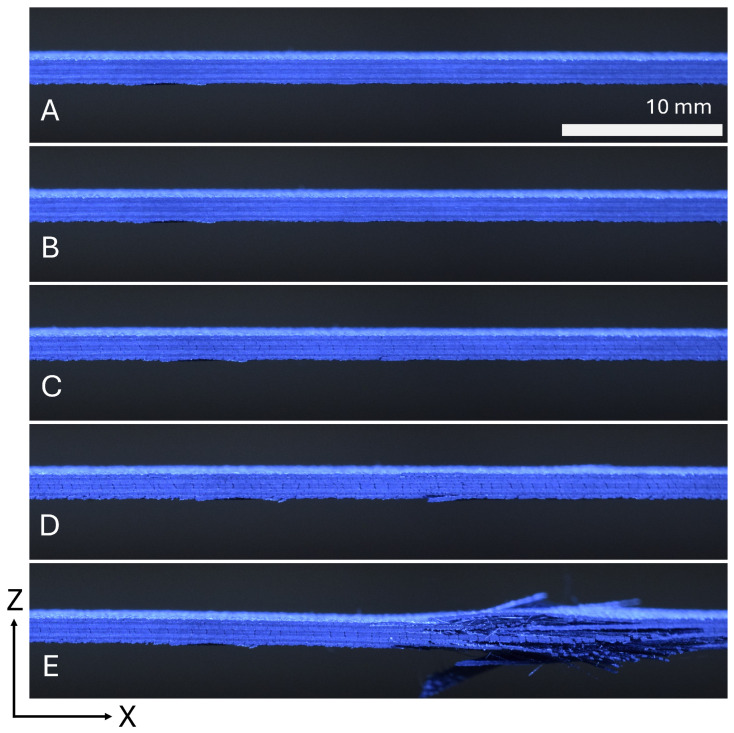
Damage progression of a PM45 tape specimen imaged from the side.

Pictures taken of the side of the specimen in Figure 9 show the cracks that form in the laminae throughout the experiment to demonstrate progressive failure. The crack density increases throughout the experiment, and delamination is seen in the failure of the specimen.

**Figure 10:**
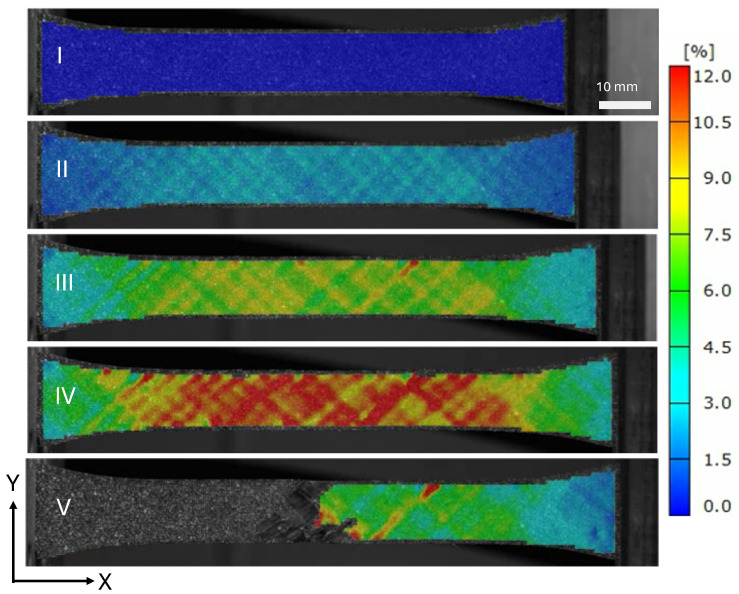
Surface loading strains in the loading direction $\epsilon_{x}$ of a woven PM45 specimen.

**Figure 11:**
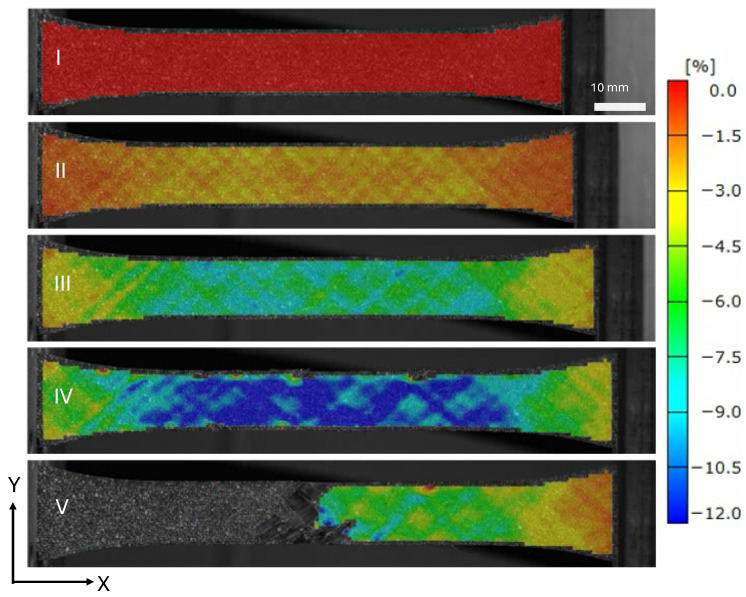
Surface loading strains in the loading direction $\epsilon_{y}$ of a woven PM45 specimen.

The strain field at the indicated locations from Figure 5 is displayed in Figure 10 and Figure 11. The saturation of micro-cracks in the structure occurred earlier than in the tape specimens and was continuous throughout the experiment. At time point II, the surface strains indicate that many cracks form internally. Through points III and IV, the strains intensify until the final failure occurs at time point V.

**Figure 12:**
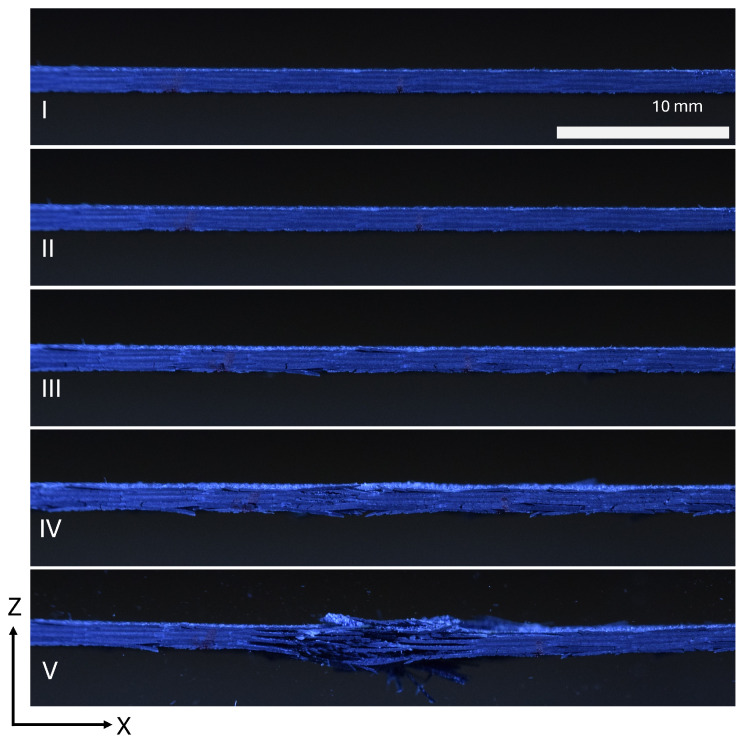
Damage progression of a PM45 woven specimen imaged from the side.

Pictures taken of the side of the specimen in Figure 12 show the cracks that form in the laminae throughout the experiment to demonstrate progressive failure. The cracks visible in the experiment are less structured than in the tape specimen. The complex architecture of the woven samples allows for damage to initiate on a much smaller scale. This occurs in the space within the tows of carbon fiber and between the tows and the matrix. These smaller-scale damaging events are smoothed out in the response, resulting in no kink in the $\epsilon_{x}$ and $\epsilon_{y}$ measurements.

#### 2.2.3. EST Fit from PM45 Experiment

**Figure 13:**
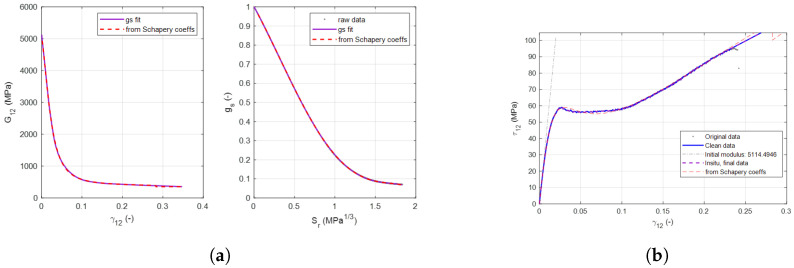
Calculation of EST degradation for M21 tape specimens. (**a**) Calculation of EST degradation for M21 tape samples. (**b**) EST recreation of tape shear strain behavior.

**Figure 14:**
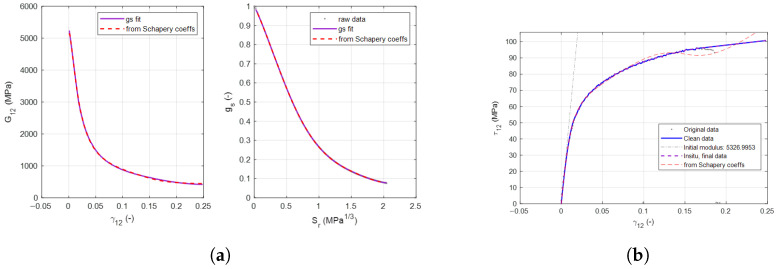
Calculation of EST degradation for M21 woven specimens. (**a**) Calculation of EST degradation for M21 woven samples. (**b**) EST recreation of woven shear strain behavior.

In order to incorporate the effects of EST into a numerical model, the Schapery polynomial coefficients and energy dissipation must be calculated from the results of the PM45 test. These curves are shown in Figure 13 and Figure 14. The decay of the shear modulus, energy dissipation, and the resultant shear stress–shear strain relationship is calculated.

The strains determined to describe the transition from the nonlinear pre-peak region to crack band occur at 0.03 for the tape specimens and 0.05 for the woven specimens.

## 3. Interface Characterization Experimental Study

In the experimental study, DCB and ENF experiments identify the mode I and mode II fracture toughnesses for all types of interface. This characterization is needed to understand how delamination is expected in hybrid laminates with various combinations of tape, woven, and hybrid interfaces.

### 3.1. Interface Characterization Specimen Description

Three samples for each of the interface types were investigated, for a total of nine DCB and nine ENF experiments. Each test specimen was 200 mm in length and 25 mm in width, with the measured thickness varying based on the laminate type described in Table 2. The artificial crack was set during the manufacturing process by including a thin Teflon film with a thickness of 0.0005 inch, covering 80 mm of the length of the specimen. These samples were made using prepreg laminae and cured in an autoclave. After curing, the samples were cut using a waterjet and the edges were inspected with a microscope to ensure there was no induced delamination in the cutting process. The edge of each specimen was painted white, with the artificial crack front and every 10 mm beyond marked with a black tally to track the growth of the crack during the experiments. These experiments were carried out on the M21 material system to investigate the cohesive parameters and fracture toughnesses of the interface-toughened material and to investigate how these values change based on the laminae adjacent to the crack.

The stacking sequences of these laminates were designed so that the B matrix calculated using the Classical Lamination Theory (CLT) produced a zero matrix for the entire laminate and for each half laminate. The interface type column can be interpreted to show the type and orientation of each lamina adjacent to the studied interface. A subscript *T* represents a unidirectional tape lamina and a subscript *W* represents a woven lamina containing tows woven in perpendicular directions. In this paper, both experiments and simulations were performed on samples A, B, and C. The detailed layup for each experimental sample is listed in Table 3. The side sample was spray-painted with matte white paint. A mark on the top surface and the side edge indicated the artificial crack boundary in the specimen, and black markings indicate every 10 mm beyond the crack front to measure crack growth during the experiments.

The design of these specimens had to consider the stiffness of each of the experiments such that they would fail before reaching a large deformation or fail in shear before enough deformation. The flexural strength and modulus depends greatly on the thickness of the laminate [35,36].

**Figure 15:**
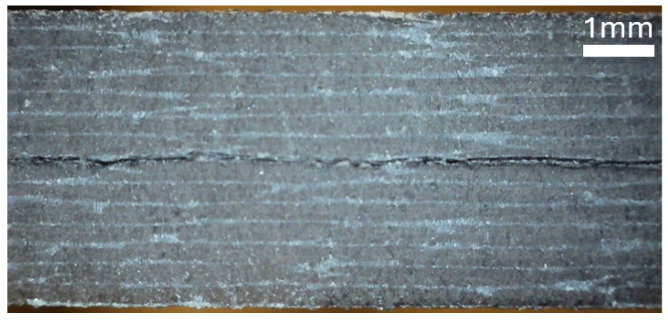
Microscope image of the side of specimen A, showing the woven pattern.

**Figure 16:**
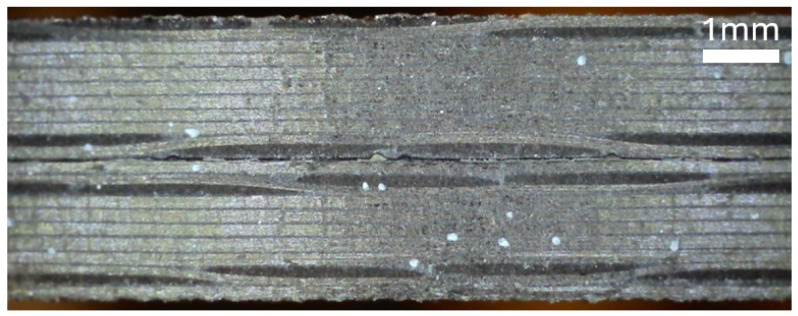
Microscope image of the side of specimen B, showing the hybrid pattern.

**Figure 17:**
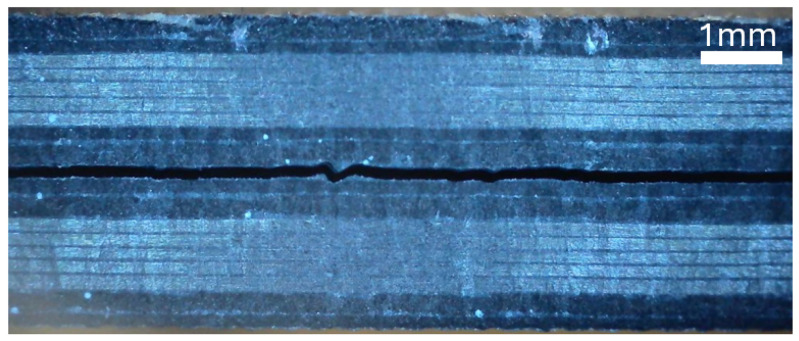
Microscope image of the side of specimen C, showing the hybrid pattern.

Images of the side profile of each laminate are shown in Figure 15, Figure 16, and Figure 17. The microscope images were taken in the region in which the interface separation film is visible in the mid-plane.

### 3.2. Double Cantilever Beam

The double cantilever beam (DCB) experiment is used to calculate the mode I fracture toughness between carbon fiber laminae.

#### 3.2.1. DCB Experimental Method

**Figure 18:**
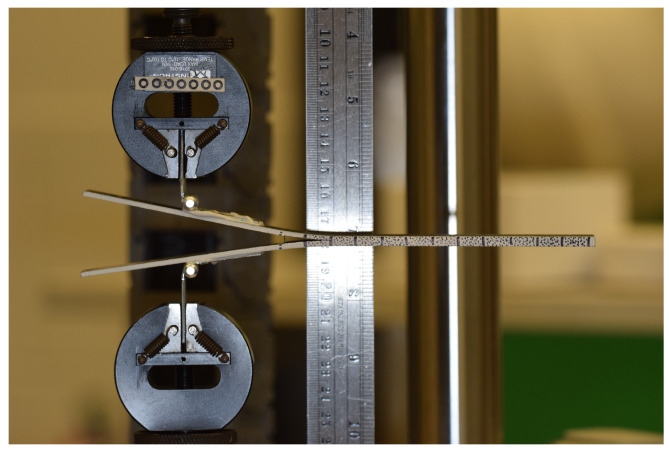
Experimental setup for the DCB experiment.

Figure 18 illustrates the concept of the experiment in which fixture clamps pull the two arms of the DCB specimen apart. Piano hinges are adhered to the unbonded arms of the specimens using a locktite quick-set epoxy to provide a pivot point on the specimen and to prevent a moment from generating at the loaded edge. The hinges were positioned such that the initial distance from the hinge to the artificial crack front was 40 mm. In this experiment, the crack propagates along the mid-plane of the specimen. The crack growth was monitored using a Nikon camera with images taken at a two-second interval. The loading rate for the DCB experiment was 10 mm/min. No machine compliance was observed in these experiments.

Each specimen was initially loaded in its non-pre-cracked (NPC) state. A small drop in the load corresponded to an observable propagation of the crack front. This was performed to create a natural crack front in the material in subsequent tests. The test specimen was then re-loaded from its initial state for the full DCB experiment as a pre-cracked (PC) specimen.

#### 3.2.2. DCB Experimental Results and Analysis

**Figure 19:**
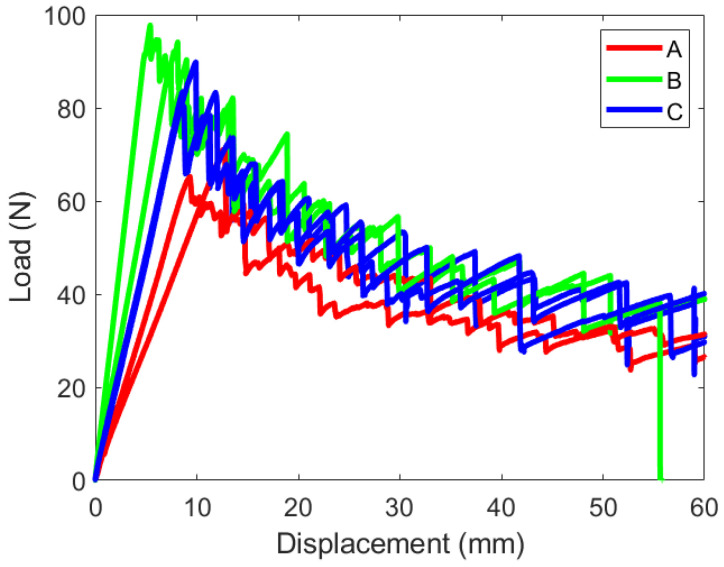
Load versus displacement response for each DCB experiment.

The load–displacement response of the DCB experiments are shown in Figure 19. These plots are for the PC configuration.

The initial stiffness of each specimen is slightly different due to differences in the starting crack length. The initial pre-crack does not produce identical starting points for the full experiment. These three layups experience unstable crack growth; an expected characteristic of woven and hybrid laminates. The load response vertically drops as the crack grows intermittently. This is different from the traditional $0_{T}\left|\right|0_{T}$ response, which produces a smooth decay where the crack grows continuously.

The mode I fracture toughness values were calculated using the novel method described in a paper by Xu and Guo [37]. This technique requires the geometric and material properties of the test specimen, the load and displacement history of the tests, and the initial and final crack lengths. The crack length during the test can be challenging to monitor due to the instability of the crack propagation. This method eliminates that requirement. The average fracture toughness values are reported in Table 4.

**Figure 20:**
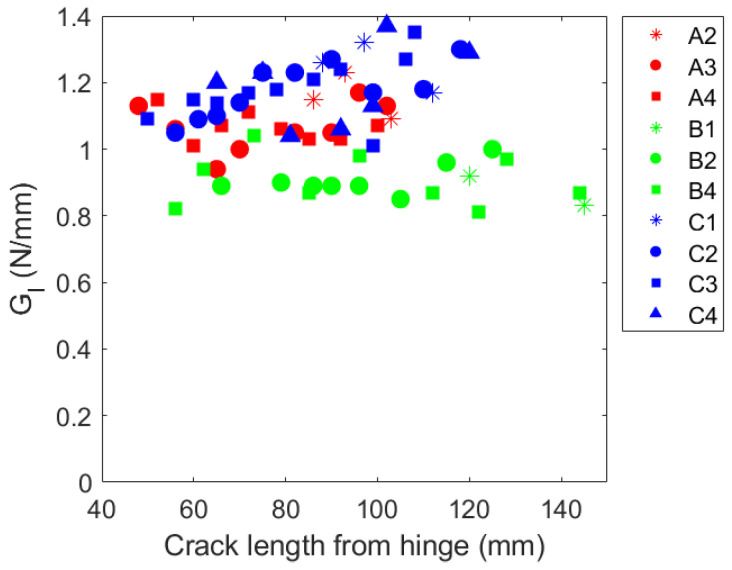
Mode I fracture toughness calculation as a function of crack length.

The measured fracture toughness values throughout the experiment are plotted against the crack length in Figure 20. Specimens A and B have consistent measurements throughout crack growth. The fracture toughness for sample C records larger values for greater crack lengths. The non-smooth crack growth contributes to fluctuations in the fracture toughness calculation, in this study, the value reported in Figure 20 uses the load value at a local peak before a sudden crack growth. This was selected because these local peaks represented where the interface could still carry the load, while the local minimum represented where the the interface was able to remain intact after the crack growth; however, at the local minimums, there is also an effect of the momentum of the crack, and thus, the local peak values with their corresponding crack lengths should be reported.

### 3.3. End-Notch Flexure

The End-Notch Flexure test is used to calculate the mode II fracture toughness between carbon fiber laminae.

#### 3.3.1. ENF Experimental Method

**Figure 21:**
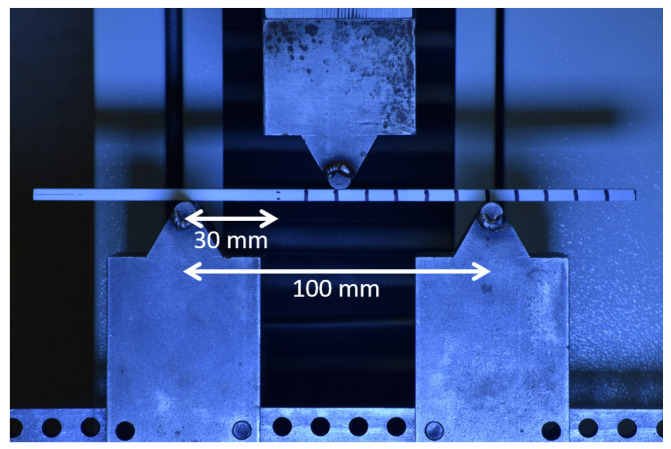
Experimental setup for the ENF experiment.

A steel three-point bend fixture was used to perform the ENF experiments. The span between the bottom roller supports was 102 mm with a top roller positioned at the center of the specimen. The diameter of each roller was 6.35 mm. The top fixture moved downward into the specimen to cause the specimen to bend, and allow the crack to propagate in shear along the sample’s mid-plane. The test was conducted based on the guidelines described in ASTM-D7905 [38] with a loading rate of 1.0 mm/min. The crack growth was monitored using a Nikon camera taking images at a two-second interval from the perspective in Figure 21. Machine compliance was observed for approximately the first 40 N of force. The initial stiffness of the specimen was calculated using data after the machine compliance region in the data, when the stiffness reached its peak value.

The shear forces accumulate at the crack front in the mid-plane and abruptly propagate through the interface. The progressive failure of these specimens during the ENF experiment allowed for the calculation of the mode II fracture toughness of the test specimens.

**Figure 22:**
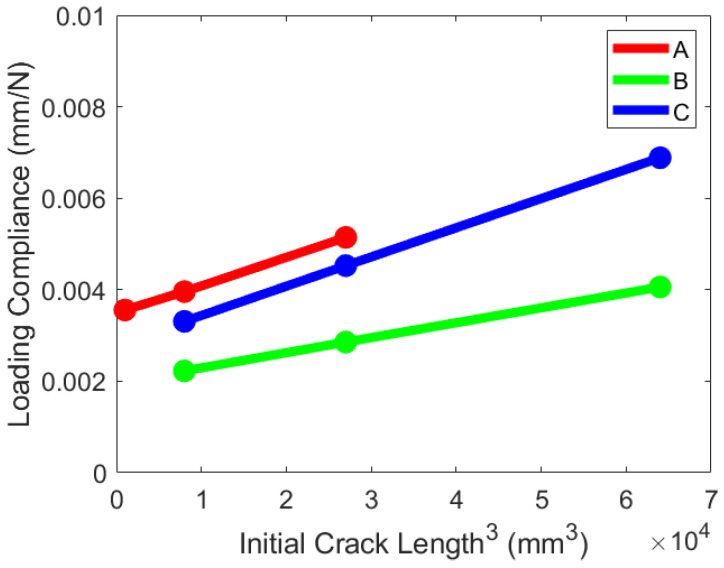
ENF specimen compliance validation.

A validation of the compliance for each specimen was performed. This was performed by placing the test specimen on the three-point bend fixture at three different positions and loading the specimen to roughly 25% of the expected failure load. The starting crack length is measured from the center of the left-end roller support and towards the centerpoint of the test specimen. In Figure 21, this value is 30mm, the nominal test value. While no load is on the test specimen, the test specimen can be shifted on the supports such that the crack front is closer to or further from the left support. This calibration generates a stiffness for each starting crack length which can be used in additional calculations for mode II fracture toughness. Plotting the compliance of each specimen against the cubed crack length from the close edge support produces a linear response as shown in Figure 22. No damage and no crack propagation were observed during the compliance validation loading steps.

Like in the DCB experiment, an initial NPC test is performed to advance the crack from the artificial insert to a natural front. The initial crack propagation was monitored for all specimens. The NPC experiment was conducted until the crack front moved at least 10 mm to ensure that there was no influence of the insert. The propagation in samples A and C was slower and more stable than specimen B. In the case of specimen B, the crack traveled 20 mm to the location of the center loading roller, and the test was stopped. The PC state experiments have the position on the rollers adjusted such that the starting crack length is identical for each experiment. An ultrasonic testing scan is performed on the specimens before testing and after the NPC and PC experiments. This measurement offers additional insight into the position of the crack internally rather than just the visible edge of the specimen.

#### 3.3.2. ENF Experimental Results and Analysis

**Figure 23:**
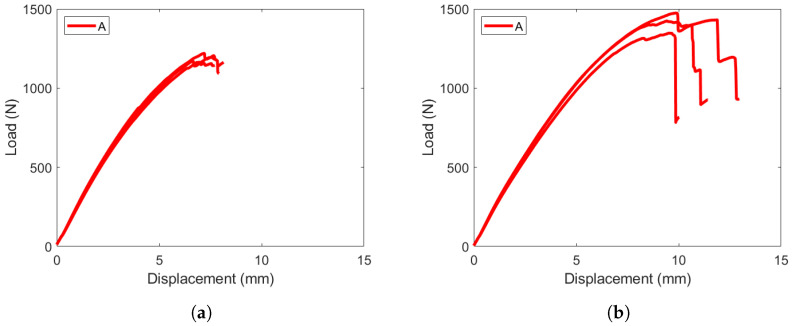
ENF load versus displacement response for layup A. (**a**) Layup A NPC response, loaded until first load drop and crack advancement. (**b**) Layup A PC response, loaded for significant crack propagation.

The test data from the ENF experiments are plotted separately. Figure 23 plots the load–displacement response for the $\pm 45_{W}\left|\right|\pm 45_{W}$ specimens. Specimen A was more flexible than B and C and required closer boundary supports to generate mode II crack growth. The PC specimens failed at higher loads than the NPC specimens. While there are multiple contributing factors to this, it is likely caused by the location the crack travels through. The NPC case sets the failure location at the tip of the artificial crack front, and the first damage growth occurs after that point. The PC experiment experiences crack propagation within the woven lamina and not directly in the interface plane, which generates higher loads in the ENF experiment.

**Figure 24:**
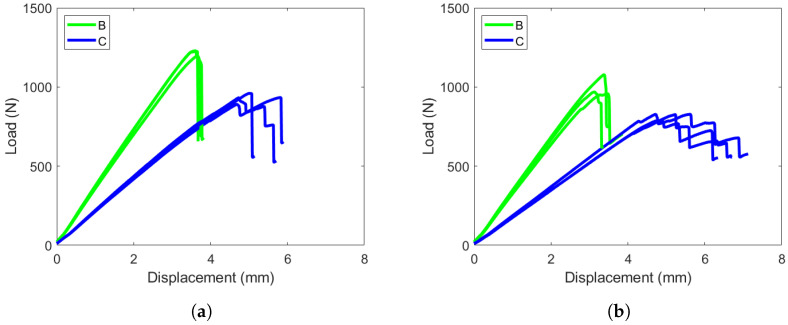
ENF load versus displacement response for layups B and C. (**a**) Layup B and C NPC responses, loaded until first load drop and crack advancement. (**b**) Layup B and C PC responses, loaded for significant crack propagation.

Specimens B and C are plotted together in Figure 24. Both hybrid interface specimens fail at lower loads and displacements. The crack propagated from its initial crack start point to the location of the top roller when failure was reached for specimen B. This event corresponds to the vertical drop in the load response of specimen B. Specimen C experienced a slower failure, and the crack grew in shorter advances corresponding to multiple load drops in the load response.

The mode II fracture toughness for each of these experiments can be calculated using the method described in ASTM D7905. The fracture toughness calculation produces a single value that represents the average fracture toughness over a heterogeneous surface. The tabulated fracture toughnesses are shown in Table 5.

**Figure 25:**
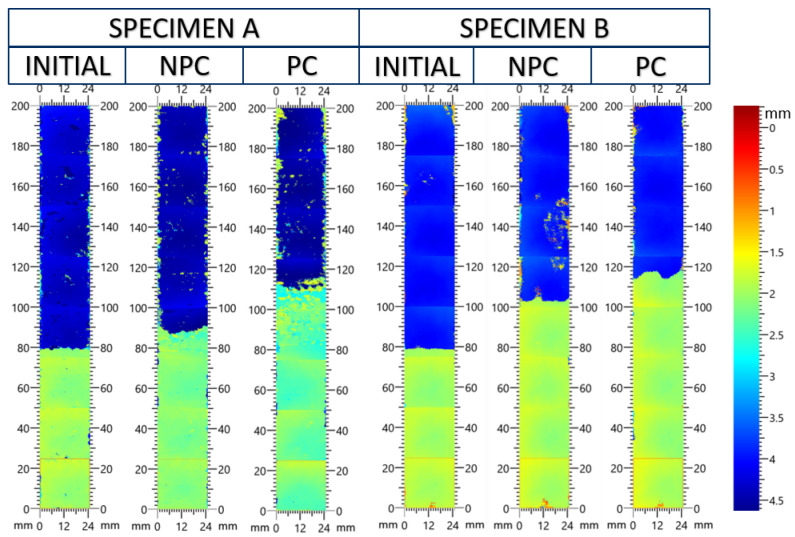
UT C-scan of the A and B type test specimens initially, after the NPC test, and after the PC test.

**Figure 26:**
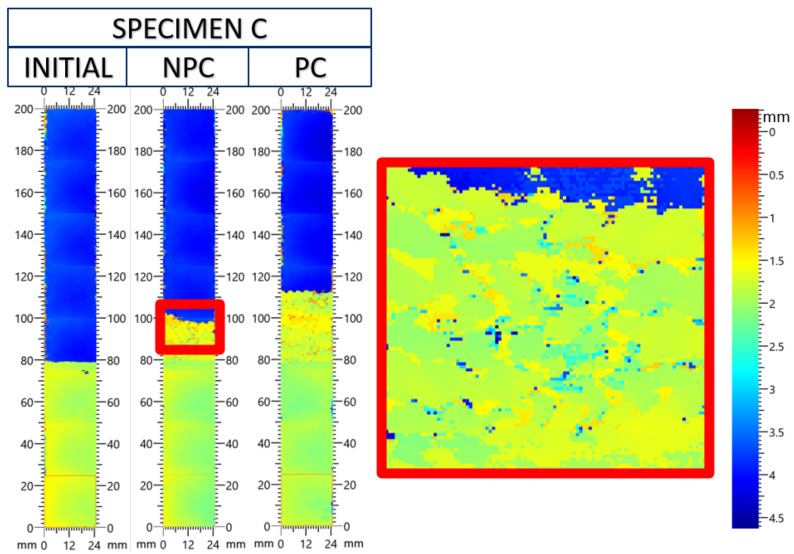
UT C-scan of the C type test specimens initially, after the NPC test, and after the PC test.

Figure 25 and Figure 26 show the development of crack growth at different stages of the ENF experiment. A color bar on the right of the figures indicates depth into the test specimen. The yellow/green region represents the mid-plane, and the dark blue region represents the bottom of the test specimen. The initial crack front was straight across the specimen and at the 80 mm mark. The next scan was performed after the NPC experiment to advance the crack from the artificial front to a natural position. In each experiment, the crack growth in the experiment increased between 10 and 20 mm before the test was stopped. The third scan was performed after the PC experiment. The crack front does not stay perfectly straight after testing. The crack front location fluctuates by 8 mm at the end of the specimen B PC experiment.

In Figure 26, a region beyond the crack front in the NPC experiment is enlarged. The calculated fracture toughness at this interface is higher than in the other samples, and in some samples, the crack jumped from the anticipated crack interface ($\pm 45_{W}\left|\right|90_{T}$) into the woven lamina. This is also captured in the UT C-scan, as the 2 × 2 twill pattern can be observed beyond the initial crack front. This suggests that the mode II fracture toughness may be greater than the calculated value in Table 5.

## 4. Interface Characterization Numerical Study

Following the experimental characterization of the DCB and ENF specimens, a numerical framework was developed to extract the cohesive properties of each laminate interface. These parameters will subsequently be used in more complex FE models, including those aimed at evaluating impact-damage behavior.

### 4.1. FE Model Features

**Figure 27:**
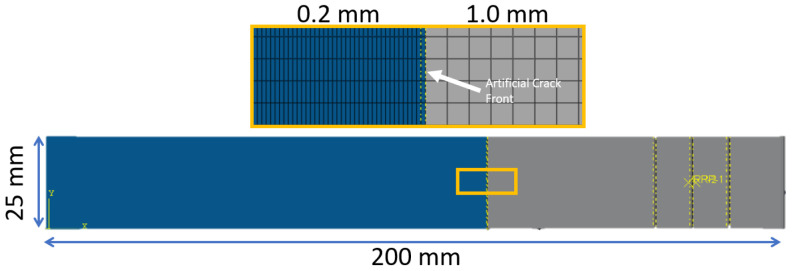
Description of the FE model used in the DCB and ENF recreations.

The finite element model (Figure 27) was developed using the commercial software Abaqus. Interface separation was modeled using the cohesive contact formulation available within Abaqus. Although alternative approaches such as cohesive elements [3] are also viable, cohesive contact was selected because it integrates more seamlessly with the impact and progressive-damage simulations planned for the later stages of this research [39,40]. Maintaining a consistent modeling strategy across studies ensures compatibility of parameters and reduces the need for re-calibration.

A locally refined mesh was used around the crack front to accurately resolve the interface behavior. Elements in the cohesive zone were assigned a size of 0.2 mm, while a coarser mesh of 1.0 mm was used ahead of the initial artificial crack. The model uses reduced integration brick elements (C3D8R), which provided computational efficiency while remaining sufficiently accurate for the global response.

The cohesive zone model utilized experimentally measured fracture toughness values for each interface type. The goal of the numerical study was to identify the corresponding cohesive strengths that reproduce the measured load–displacement response. Following prior work [41], a cohesive penalty stiffness of $10^{5}$ N/mm was applied to avoid artificial compliance while preventing numerical instability.

### 4.2. Numerical Results

Cohesive strength values were identified using a parametric study conducted in Nodeworks [42]. The design space was constructed using a Latin hypercube sampling strategy to efficiently span the range of feasible mode I ($\sigma_{c}$) and mode II ($\tau_{c}$) cohesive strengths. For each interface, between 12 and 18 simulations were executed to produce an initial response surface. The primary output metrics were the load level and displacement at the onset of crack propagation.

**Figure 28:**
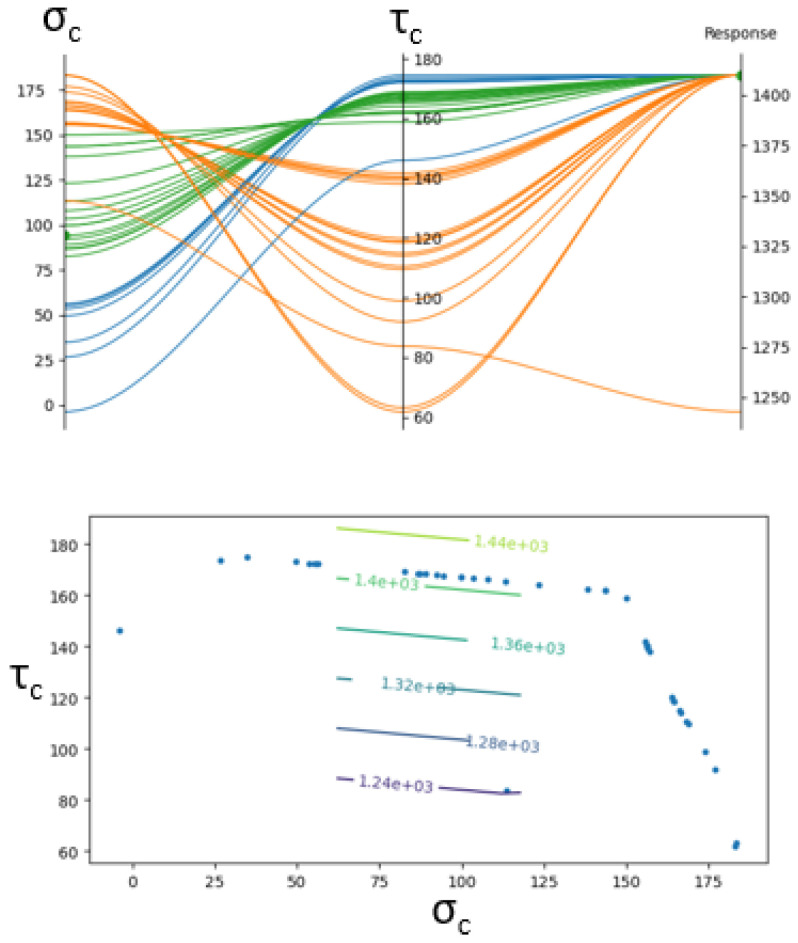
Example of optimization of cohesive parameters to meet a specified output.

Interpolation of the response surface allowed untested input combinations to be mapped to predicted failure loads. A general optimizer was then used to identify sets of cohesive strengths lying on the Pareto front—i.e., parameter combinations that most closely reproduce the experimental failure point. An example result is shown in Figure 28. The upper plot illustrates feasible ($\sigma_{c}$, $\tau_{c}$) pairs, while the lower plot highlights the relative sensitivity of the model. Notably, the optimization was strongly governed by the mode II strength, consistent with the shear-dominated nature of the ENF test. Across all interfaces, the optimal mode I strength was consistently near 100 MPa, while the calibrated mode II strength varied depending on the interface architecture.

**Figure 29:**
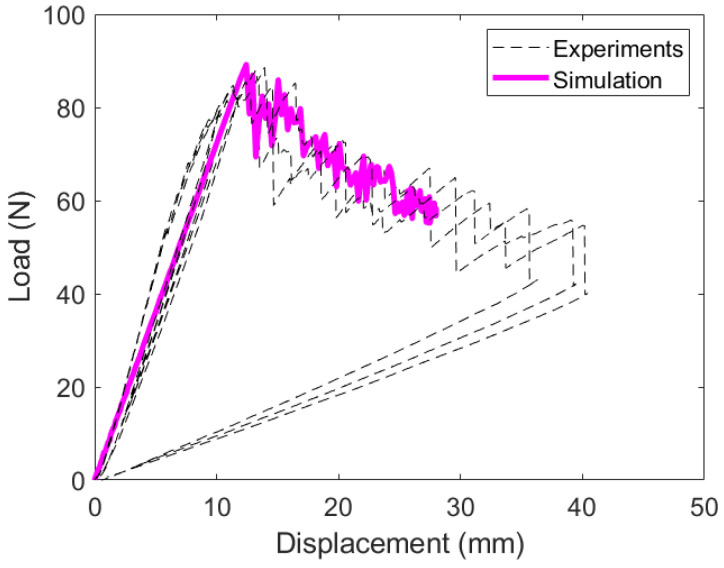
FE model validation with DCB results for a $0_{W}\left|\right|0_{W}$ interface laminate.

The optimized parameters were used to reproduce the DCB load–displacement response, as shown in Figure 29. The model accurately captures both the initial failure load and the subsequent softening behavior. Importantly, a single calibrated parameter set provided consistent predictions across all experimental repetitions, indicating robustness of the extracted cohesive strengths.

**Figure 30:**
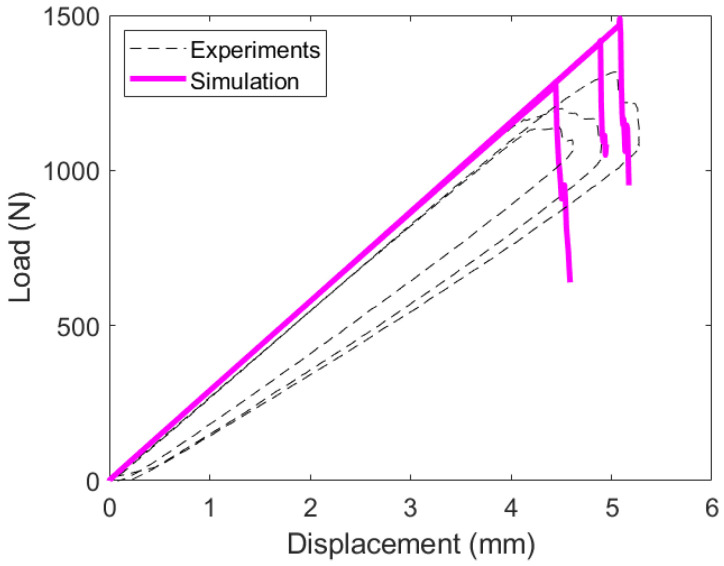
FE model validation with ENF results for a $0_{W}\left|\right|0_{W}$ interface laminate.

Recreation of the ENF experiments is shown in Figure 30. The ENF response exhibits pronounced snap-back and load drop behavior, necessitating multiple simulations per specimen. For each ENF test, fracture toughness was recalculated based on the measured unique crack propagation event. Using these specimen-specific toughness values, combined with the optimized cohesive strengths, the FE model successfully reproduced the key features of each experimental response. This agreement reinforces confidence in both the calibrated cohesive strengths and the use of mean fracture toughness values for future simulations.

The following Table 6 lists additional interface combinations from a prior study [28]. They are described for comparison with the layups tested in this study in Table 7. The fracture toughness data is recorded from the prior study and the numerical parameters were determined for all sample types using the method described in this article.

The reported data in Table 7 clearly demonstrates the higher experimental fracture toughnesses and higher numerical cohesive strengths for the woven and hybrid M21 systems and lower values for the tape M21 system. The mode I cohesive strength is a similar value for all configurations, and the mode II strength is higher as well for the woven and hybrid systems.

## 5. Conclusions

This study investigated the shear nonlinearity of both tape and woven specimens of the M21 system. Additionally, the interfacial fracture toughness and numerical cohesive strength was calculated through experimental analysis and FE analysis for tape, woven, and hybrid M21 systems. The following conclusions can be made:

- The shear response of the M21 system was characterized from PM45 experiments using EST. These results enable the nonlinearity of the M21 material response to be extracted, which assists high fidelity numerical models using this material.
- Experiments were performed to identify the mode I and mode II fracture toughness for different interfaces of the M21 composite. Six specimen types were modeled to identify the pair of cohesive strengths and toughnesses. Table 7 lists the cohesive parameters along with the experimentally determined fracture toughnesses. The results show that the failure initiation is nearly insensitive to mode I cohesive strengths and that the mode II strength can be adjusted to capture crack propagation in the DCB and ENF experiments. Higher mode II cohesive strengths are identified for the interface between two woven lamina, and slightly lower strengths are identified for hybrid interfaces. This is not surprising considering the locally undulating crack propagation path. Table 7 can be used to aid future modeling efforts of hybrid laminates that contain a mixture of tape and woven laminae.

## Figures and Tables

**Table 1:** PM45 specimen layups and thicknesses.

Sample	Layup	Thickness (mm)
Tape	[45/−45/45/−45]_s_	1.54
Woven	[$\pm 45_{W}$/$\pm 45_{W}$]_s_	1.28

**Table 2:** Interface characterization specimens.

Sample	Interface Type	Thickness (mm)
*A*	$\pm 45_{W}\left|\right|\pm 45_{W}$	4.18
*B*	$0/90_{W}\left|\right|0_{T}$	3.90
*C*	$\pm 45_{W}\left|\right|90_{T}$	3.86

**Table 3:** Interface characterization layups.

Sample	Layup
*A*	[${\left(\pm 45_{W}\right)}_{7}\left|\right|{\left(\pm 45_{W}\right)}_{7}$]
*B*	[$0/90_{W},\;{\left(0_{T}\right)}_{7},\;0/90_{W}\left|\right|0_{T},\;0/90_{W},\;{\left(0_{T}\right)}_{5},\;0/90_{W},\;0_{T}$]
*C*	[$\pm 45_{W},\;90_{T},\;{\left(0_{T}\right)}_{5},\;90_{T},\;\pm 45_{W}\left|\right|90_{T},\;\pm 45_{W},\;{\left(0_{T}\right)}_{5},\;\pm 45_{W},\;90_{T}$]

**Table 4:** Calculated mode I fracture toughness values for each layup.

Sample	$G_{\mathrm{Ic}}$ (N/mm)
*A*	1.08
*B*	0.90
*C*	1.20

**Table 5:** Calculated mode II fracture toughness values for each layup.

Sample	$G_{\mathrm{IIc}}$ (N/mm)
*A*	2.48
*B*	1.76
*C*	3.10

**Table 6:** Additional interface characterization specimens from prior studies.

Sample	Interface Type
*D*	$0/90_{W}\left|\right|0/90_{W}$
*E*	$\pm 45_{W}\left|\right|0/90_{W}$
*F*	$\pm 45_{W}\left|\right|0_{T}$
*G*	$0_{T}\left|\right|0_{T}$

**Table 7:** M21 interface cohesive parameters.

Sample	Type	$\sigma_{c}$ (MPa)	$\tau_{c}$ (MPa)	$G_{\mathrm{Ic}}$ (N/mm)	$G_{\mathrm{IIc}}$ (N/mm)
*A*	Woven	106	198	1.08	2.48
*B*	Hybrid	90	140	0.90	1.76
*C*	Hybrid	95	146	1.20	3.10
*D*	Woven	94.3	168	1.01	2.19
*E*	Woven	101	199	1.14	2.34
*F*	Hybrid	95.8	142	1.13	1.80
*G*	Tape	100	120	0.254	0.91

## Data Availability

The data presented in this study are available on request from the corresponding author due to research sponsors’ ownership of the data reported in the study.
